# Effects of Cyclic Chronic Heat Stress on the Expression of Nutrient Transporters in the Jejunum of Modern Broilers and Their Ancestor Wild Jungle Fowl

**DOI:** 10.3389/fphys.2021.733134

**Published:** 2021-10-21

**Authors:** Nedra Abdelli, Alison Ramser, Elizabeth S. Greene, Lesleigh Beer, Travis W. Tabler, Sara K. Orlowski, José Francisco Pérez, David Solà-Oriol, Nicholas B. Anthony, Sami Dridi

**Affiliations:** ^1^Animal Nutrition and Welfare Service, Department of Animal and Food Sciences, Universitat Autònoma de Barcelona, Bellaterra, Spain; ^2^Department of Poultry Science, University of Arkansas, Fayetteville, AR, United States

**Keywords:** broilers, heat stress, glucose transporters, amino acid transporters, fatty acid transporters

## Abstract

Heat stress (HS) has been reported to disrupt nutrient digestion and absorption in broilers. These effects may be more prominent in fast-growing chickens due to their high metabolic activity. However, the underlying molecular mechanisms are not yet fully elucidated. Hence, the current study aimed to evaluate the effect of chronic HS on jejunal nutrient transport in slow- (Athens Canadian Random Bred, ACRB from 1950), moderate- (The 1995 random bred, 95RAN), rapid- (modern broilers, modern random bred, MRB) growing birds and their ancestor wild jungle fowl (JF). One-day male chicks (*n* = 150/line) were placed by line in environmentally controlled chambers and kept under the same industry-standard environmental conditions until d28. On d29, an 8-h daily cyclic HS (36°C) was applied to half of the chambers, which lasted until d55, while keeping the rest under thermal neutral (TN, 24°C) conditions. Jejunum tissues were collected for morphology assessment and molecular analysis of carbohydrate-, amino acid-, and fatty acid-transporters. MRB exhibited the highest body weight (BW) followed by 95RAN under both conditions. HS decreased feed intake (FI) in MRB and 95RAN, which resulted in lower BW compared to their TN counterparts; however, no effect was observed in ACRB and JF. MRB showed a greater villus height (VH) to crypt depth (CD) ratio under both environmental conditions. Molecular analyses showed that glucose transporter (GLUT) 2, 5, 10, and 11 were upregulated in MRB compared to some of the other populations under TN conditions. HS downregulated GLUT2, 10, 11, and 12 in MRB while it increased the expression of GLUT1, 5, 10, and 11 in JF. GLUT2 protein expression was higher in JF compared to ACRB and MRB under TN conditions. It also showed an increase in ACRB but no effect on 95RAN and MRB under HS conditions. ACRB exhibited greater expression of the EAAT3 gene as compared to the rest of the populations maintained under TN conditions. HS exposure did not alter the gene expression of amino acid transporters in MRB. Gene expression of CD36 and FABP2 was upregulated in HS JF birds. Protein expression of CD36 was downregulated in HS JF while no effect was observed in ACRB, 95RAN, and MRB. Taken together, these data are the first to show the effect of HS on jejunal expression of nutrient transporters in three broiler populations known to represent 70 years of genetic progress in the poultry industry and a Red Jungle Fowl population representative of the primary ancestor of domestic chickens.

## Introduction

Heat stress (HS) is considered one of the main threats to the poultry industry especially under continuous climate changes, including global warming (Perini et al., 2021). The high susceptibility of chickens to HS is attributed to their limited ability to regulate heat loss due to the presence of feathering and the lack of sweat glands (Wasti et al., 2020). On the other hand, the genetic selection of fast-growing meat-type broilers during the last few decades has focused narrowly and intensely on increasing growth rates and meat yield, improving feed efficiency, and decreasing slaughter age (Wasti et al., 2020). Indeed, modern broilers are characterized by higher metabolic activities compared to their ancestors, resulting in increased body heat production (Al-zghoul et al., 2019). Therefore, the positive achievements in terms of growth performance were accompanied by higher sensitivity in the gut to HS (Havenstein et al., 2003; He et al., 2018a), a lack of effective thermoregulation and heightened metabolism, entailing a strenuous adaptation to harsh environments, and difficulties to cope with HS (Perini et al., 2021).

Heat stress occurs when heat load of an animal is greater than its capacity to lose heat and may cast a shadow over the performance and profitability of livestock production. In this sense, a growing body of scientific evidence has documented the detrimental effects of HS on broiler growth, metabolism, and physiology, such as impairing the intestinal morphology by shortening the villus and deepening the crypt (Song et al., 2017; He et al., 2018a), decreasing jejunal weight and length (Garriga et al., 2006), impairing the intestinal integrity, and inducing oxidative stress leading to epithelial damage and inflammatory response (Lian et al., 2020). It also possesses a negative impact on the immune system (Cui et al., 2016) by triggering the expression of heat shock proteins (HSPs), and thereby leading to decreased energy metabolism (Liu et al., 2015), which reduces the growth performance. The impaired poultry productivity by HS was also attributed to the reduced utilization of nutrients, resulting from altering the activities of enzymes, such as amylase, maltase, lipase, trypsin, and chymotrypsin (Song et al., 2017; He et al., 2018a) and modulating the expression of the genes responsible for nutrient transport (Goel et al., 2021). The abovementioned negative effects may depend on both temperature and duration of HS exposure (Goel et al., 2021). In this context, some studies reported a reduced glucose uptake under chronic HS as evidenced by the downregulation of the sodium-dependent glucose transporter (SGLT) gene responsible for the glucose absorption and the glucose transporter 2 (GLUT2) gene, involved in the transfer of up-taken fructose and glucose into portal blood capillaries, in the intestine of broiler chickens (Sun et al., 2015; Habashy et al., 2017a; Al-zghoul et al., 2019). Similarly, protein expressions of SGLT1, GLUT1, and GLUT10 were lowered in the intestine of laying hens subjected to HS for 12 weeks (Orhan et al., 2019). HS has been also shown to impair fatty acid transporters (FATs), especially by reducing the gene expression of fatty acid-binding proteins (FABPs), such as FABP1, irrespective of exposure time and intensity of stress (Sun et al., 2015; Habashy et al., 2017b; Al-zghoul et al., 2019), the gene expression of fatty acid transport proteins (FATPs), and the gene and protein expression of the cluster of differentiation 36 (CD36) (Al-zghoul et al., 2019; Orhan et al., 2019). Research has shown conflicting results regarding the effects of chronic HS on amino acid transport. Although a decrease of PEPT1 has been reported in broilers subjected to 35°C for 19 days (Habashy et al., 2017b) and laying hens exposed to 34 ± 2°C for 8 h/day during 12 weeks (Orhan et al., 2019), other authors have observed no variation of this transporter in broilers exposed to 32°C for 7 days (Sun et al., 2015). In spite of its detrimental effects on nutrient digestibility and absorption, HS was not shown to modulate *SLC7A1* and *SLC7A7* gene expression (Sun et al., 2015; Yi et al., 2016; Song et al., 2017; He et al., 2018b), but it rather enhances amino acid catabolism in broiler intestine (Lara and Rostagno, 2013).

Although the aforementioned studies are seminal and provided valuable insights, they used only modern chickens (broilers or layers). In fact, there is still a lack of clear understanding of how the gastrointestinal tract has changed in response to intensive genetic selection for rapid growth and feed efficiency and how is it affected by HS. The present study was performed to evaluate the effects of chronic HS on the gene and protein expression of carbohydrate, amino acid, and FATs in the jejunum of three broiler populations [slow growing 1950 (Collins et al., 2016), moderate growing 1995 (Harford et al., 2014), and modern fast-growing 2015 (Orlowski et al., 2017)] known to represent 70 years of broiler genetic progress, and the jungle fowl (JF), representative of the ancestral origin of the modern broiler (Orlowski et al., 2017).

## Materials and Methods

### Chicken Populations

The current study involved four research lines that are housed and maintained at the University of Arkansas research farm. The first line represents the commercial broiler chicken of the 1950s (Athens Canadian Random Bred, ACRB) characterized by slow growth (Collins et al., 2016). One thousand nine hundred ninety-five random bred (95RAN), a moderate-growing line consisting of seven parent stock male and six parent stock female lines commercially available in the 1990s (Harford et al., 2014). The third line is a modern random bred (MRB) population initially established in 2015 at the University of Arkansas that originally consisted of four commercially available broiler packages from three different broiler genetics companies; Cobb MX × Cobb 500, Ross 544 × Ross 308, Ross Yield 1 × Ross 708, and the Hubbard HiY package. The four packages have been blended homogenously after five generations of random mating to create a population representing a commercially available broiler from 2015 (Orlowski et al., 2017). The fourth generation is the South East Asian JF, serving as the common ancestor to the commercial broiler (Orlowski et al., 2017).

All populations are maintained at the University of Arkansas research farm under close care and supervision and are randomly mated each generation with the avoidance of full and half-sibling pairings. The study was conducted in accordance with the recommendations in the guide for the care and use of laboratory animals of the National Institutes of Health and the protocols received prior approval from the University of Arkansas Animal Care and Use Committee under protocols 18,083 and 16,084.

### Experimental Design, Environmental Exposure, and Animal Husbandry

Day-old broiler chicks from the four chicken lines hatched at the University of Arkansas were vent-sexed and individually wing-banded with a number and barcode. Male chicks were separated by line and housed in 12 environmentally controlled chambers in the Poultry Environmental Research Laboratory at the University of Arkansas. Each chamber consists of two equally sized pens allowing for triplication of a 4 × 2 factorial design. Twenty-five male chicks of the same line were randomly placed in each pen and kept at an approximate density of 0.5 m^2^ per bird in all pens. All birds were allowed *ad libitum* access to feed and fresh water. The lighting program was set to 23L: 1D (L: light; D: darkness) during the first week and 20L: 4D for the remainder period (day 8–56) of the trial. Chickens were given a two-phase feeding program consisting of commercially available starter (day 0–28) and finisher (day 29–55) formulated to meet or exceed National Research Council (NRC) recommendations (National Research Council, 1994).

The brooder temperature was maintained at 32°C during the first 3 days and was then gradually reduced to 31°C on days 4–6, 29°C on days 7–10, 27°C on days 11–14, and 25°C for day 15 through day 28. The environmental treatments began the morning of day 29 by keeping half of the chambers in a TN environment at a constant temperature of 25°C for the remainder of the study, whereas the remaining six chambers were subjected to an 8 h daily cyclic HS (36°C) from 8 a.m. to 4 p.m., until processing (Tabler et al., 2020). This design resulted in three pens (75 birds) per line being subjected to either a TN or HS environment.

### Growth Performance Evaluation and Sample Collection

Live BW and FI were recorded weekly. At the end of the experiment, six birds/line/environmental treatments were randomly selected, electrically water bath stunned (11V, 11 mA, 10), and manually cut through the left carotid artery and allowed to completely bleed. Samples from the jejunal tissue were collected and kept in 4% paraformaldehyde in phosphate-buffered saline (PBS) for further analysis of intestinal morphology. Segments from the jejunum were harvested for molecular analyses as described below. The tissues were snap frozen in liquid nitrogen and stored at −80°C until analysis.

### Intestinal Morphology Analysis

Jejunum samples collected at 55 days of age were embedded in paraffin using a tissue processor. Sections of 2.5 μm were stained with hematoxylin and eosin before being analyzed with a digital microscope camera (Leica DFC450 C; Leica Microsystems Ltd.) and the ViewPoint Light software version 1.0.0.9628. Images were analyzed by the same person. The morphometric variables measured included VH, CD, and VH to relative CD ratio (VH: CD ratio). Twelve villi were measured for each sample and only complete and vertically oriented villi were evaluated. The mean from 12 villi per sample was used as the mean value for further analysis.

### Cell Culture

As no established chicken jejunal cell line currently exists, the non-transformed intestinal porcine epithelial cells from the jejunum (IPEC-J2) were selected as an *in vitro* model in this study and has been used in previous chicken studies. The IPEC-J2 cell line was originally derived and characterized from jejunal epithelia of unsuckled piglets (Schierack et al., 2006). The cells of the present study were a kind gift from Dr. Maxwell C. (University of Arkansas). Cells were cultured in six-well-plates with 10% fetal bovine serum (FBS) and 1% penicillin/streptomycin (Life Technologies, Carlsbad, CA, USA) in Dulbecco's Modified Eagle Medium (DMEM)/F-12/HAM (Life Technologies, Carlsbad, CA, USA) at 37°C in a humidified atmosphere of 5% CO_2_ and 95% air. At ~80–90% confluence, cells were exposed to HS (45°C) for 2 h. Cells maintained at 37°C were used as controls.

### RNA Isolation, Reverse Transcription, and Quantitative Real-Time PCR

One microgram of total RNA was extracted from chicken jejunum tissues or IPEC-J2 cell line by trizol reagent (Thermo Fisher Scientific, Rockford, IL) following the recommendations of the manufacturer. For each sample, total RNA concentration was determined using Take 3 Micro Volume Plate and the Synergy HT Multi-Mode Microplate Reader (BioTek, Winooski, VT, USA). RNA quality and integrity were assessed using the ratio of absorbance (260/280) and 1% agarose gel electrophoresis. Afterward, RNAs were treated with DNAse and reverse transcribed *via* qScript cDNA SuperMix (Quanta Biosciences, Gaithersburg, MD, USA). The cDNA was then amplified by real-time quantitative PCR (Applied Biosystems 7500 Real-Time PCR system) with Power SYBR Green Master Mix (Thermo Fisher Scientific, Rockford, IL, USA) as described previously (Greene et al., 2020; Tabler et al., 2020; Emami et al., 2021).

Oligonucleotide primers specific for chicken nutrient transport-related genes were used as described in Table 1. The quantitative PCR (qPCR) cycle parameters comprised the following phases: 50°C for 2 min, 95°C for 10 min followed by 40 cycles of a two-step amplification program (95°C for 15 s and 58°C for 1 min). At the end of the amplification, melting curve analysis was applied using the dissociation protocol from the Sequence Detection system to exclude contamination with unspecific PCR products. Agarose gel was used to confirm the PCR products, which showed only one specific band of the predicted size. For negative controls, no real-time products were used as templates in the qPCR and verified by the absence of gel-detected bands. Relative expressions of target genes were determined by the 2^−ΔΔCt^ method (Schmittgen and Livak, 2008). The 18S ribosomal RNA was used as an internal control to which the fold changes in gene expression were normalized. Samples extracted from JF at TN conditions were used as a calibrator.

**Table 1:** Oligonucleotide real-time qPCR primers.

**Gene**	**Accession number^a^**	**Primer sequence (5^′^ → 3^′^)**	**Orientation**	**Product size (bp)**
GLUT1	NM_205209.1	TCCTGATCAACCGCAATGAG	Forward	60
		TGCCCCGGAGCTTCTTG	Reverse	
GLUT2	NM_207178.1	GAAGGTGGAGGAGGCCAAA	Forward	61
		TTTCATCGGGTCACAGTTTCC	Reverse	
GLUT3	NM_205511.1	TTGGGCGCTTCATTATTGG	Forward	68
		CTCACTGATGTACATGGGAACAAAG	Reverse	
GLUT5	XM_040689119.1	CCTCAGCATAGTGTGTGTCATCATT	Forward	62
		GGATCGGACTGGCTCCAA	Reverse	
GLUT8	XM_040648927.1	GCTGCCTCAGCGTGACTTTT	Forward	58
		AGGGTCCGCCCTTTTGTT	Reverse	
GLUT9	XM_040670183.1	CAGTGGATGAAAGCACCTTGAC	Forward	63
		CACCGATGGCAAAAATGGA	Reverse	
GLUT10	XM_040688610.1	AACGCAGAACAAAGATTCCTGAA	Forward	65
		GTCATTCCACGTGCCAGCTT	Reverse	
GLUT11	NM_001347709.1	CCCTCATCCAGCTCATGATTCT	Forward	67
		CCACGGTCAATCAAGAGGTATCT	Reverse	
GLUT12	XM_040667840.1	TTTGTGGACCTGTTTCGTTCAA	Forward	61
		GCGTGAGCCCTACCAGCAT	Reverse	
GLUT14	XM_040672375.1	TTGGGCGCTTCATTATTGG	Forward	68
		CTCACTGATGTACATGGGAACAAAG	Reverse	
SGLT1	NM_001293240.1	AGCATTTCAGCATGGTGTGTCT	Forward	64
		TGCTCCTATCTCAGGGCAGTTC	Reverse	
SLC1A4	XM_040666934.1	CGACTGATGAACAACGCAGAA	Forward	119
(ASCT1)		TCGCCAACCTCCGCATT	Reverse	
SLC3A1	XM_040667709.1	CCTGGGCTGTGAGAAACCAA	Forward	63
(NBAT)		GGCACAAATTGAGTAGGAAGAAGAG	Reverse	
SLC6A14	XM_040670974.1	GCTTCCGTGGTCAGATTGCT	Forward	66
		TCATTTACGAGGCGTGTTTTACTG	Reverse	
SLC6A19	XM_040663289.1	CGCTGGTGTGCCTAGTTTGA	Forward	64
		ACAGCAATTTCTGATGGCTTTG	Reverse	
SLC6A20	XM_418798.6	GCTGTCAAACCCCAAAACGT	Forward	63
(SIT1)		CCCAGACCCAGTGAGAAGAAGA	Reverse	
SLC7A1	NM_001145490.1	AAAACTCCAGTAATTGCAACAGTGA	Forward	68
(CAT1)		AAGTCGAAGAGGAAGGCCATAA	Reverse	
SLC7A2	NM_001199102.1	AGCTCTCCATCCACCATGTTG	Forward	58
(CAT2)		CCAGGCACCGAACAAAGGT	Reverse	
SLC7A6	XM_040681081.1	CACGTGGGTGGCTTTGC	Forward	61
(Y + LAT2)		GAATTCTCCACGGCTCTGAACT	Reverse	
SLC7A9	NM_001199133.1	GCTGTGGGTCCTTGTTTAACCA	Forward	60
(BAT1)		TGCACCTAGTGTTGCCAGAACT	Reverse	
SLC7AL	XM_040665181.1	GCTGAGTTGGGAGCATCCA	Forward	66
		ACCAAACGCTTCCAGGATGT	Reverse	
SLC38A2	NM_001305439.1	TGGCATCCTGGGACTTTCC	Forward	66
(SNAT2)		AGCAGGAGTATCACAAAAAGAGCAA	Reverse	
LAT1	NM_001030579.2	CTGCTGCCGCCTGAGAA	Forward	62
		CGCCGGCAGGAATTCC	Reverse	
EAAT3	XM_424930.7	GGTGAAGGCGGACAGGAA	Forward	68
		TGCTGAGCAGGAGCCAGTT	Reverse	
PepT1	NM_204365.1	GACAACTTTTCTACAGCCATCTACCA	Forward	65
		CCCAGGATGGGCGTCAA	Reverse	
PepT2	NM_001319028.1	TGAAAAACCGCTCCCATCA	Forward	61
		TGTTCCGATGCCCAGTCAA	Reverse	
iFABP	NM_001007923.1	CGTACCATCGACATCGAATTCA	Forward	61
		TCCCGTCAGCCAGACTGTATT	Reverse	
CD36	XM_040686380.1	ACTGCGCTTCTTCTCCTCTGA	Forward	68
		TCACGGTCTTACTGGTCTGGTAAA	Reverse	
18s	AF173612	TCCCCTCCCGTTACTTGGAT	Forward	60
		GCGCTCGTCGGCATGTA	Reverse	

*GLUT, glucose transporter; qPCR, quantitative PCR*.

### Western Blot Analysis

Jejunum samples or IPEC-J2 cell lines were homogenized in lysis buffer (10 mmol/L Tris base, pH 7.4; 150 mmol/L NaCl; 1 mmol/L EDTA; 1 mmol/L EGTA; 0.1% Triton X-100; 0.5% Nonidet P-40; and protease and phosphatase inhibitors) and stainless-steel beads, using the Bullet Blender Storm (NextAdvance, Averill Park, NY, USA). Total protein concentrations were determined using a Bradford assay kit (Bio-Rad, Hercules, CA, USA) and run in 4–12% gradient Bis-Tris gels (Life Technologies, Carlsbad, CA, USA) and then transferred to polyvinylidene difluoride membranes. Once transferred, membranes were blocked using a Tris-buffered saline (TBS) with 5% non-fat milk and Tween 20 (TBST) at room temperature for 1 h. The membranes were washed with TBST and then incubated with primary antibodies (dilution 1:1,000) overnight at 4°C. Primary antibodies used were rabbit anti-GLUT1, rabbit anti-GLUT2, rabbit anti-GLUT3, rabbit anti-SLC38A3, and rabbit anti-CD36 (ABClonal, Woburn, MA, USA). After another wash, secondary antibodies diluted to 1:5,000 were added to 5% non-fat milk in TBS and Tween 20 and incubated with the membranes at room temperature for 1 h. The protein signals were visualized using chemiluminescence (ECL Plus; GE Healthcare, Pittsburg, PA, USA), and images were captured using the FluorCHem M MultiFluor System (ProteinSimple, San Jose, CA, USA). Prestained molecular weight marker (precision plus protein dual color) was used as a standard (Bio-Rad, Hercules, CA, USA). Protein loading was assessed by quantification of the universally expressed 70 kDa band from staining with universal protein Ponceau S (PS) stain (G-Biosciences, St. Louis, MO, USA) (Sander et al., 2019). Image acquisition and analysis were performed by AlphaView software (version 3.4.0, 1993–2011; ProteinSimple). One representative blot was shown for each protein.

### Immunofluorescence

Immunofluorescence staining was performed as previously described (Dridi et al., 2012; Greene et al., 2020). Briefly, IPEC-J2 cells were grown in chamber slides, exposed to 37 or 54°C as described above and fixed with methanol for 10 min at −20°C before being permeabilized with Triton-X 100. Cells were blocked with serum-free protein block (Dako, Carpinteria, CA, USA) for 1 h at room temperature, then incubated with anti-GLUT1, anti-GLUT2, anti-GLUT3, anti-HSP60 (Santa Cruz Biotechnology, Dallas, TX, USA), or anti-HSP70 (Pierce Thermo Scientific, Rockford, IL, USA). All antibodies were diluted at 1:200, in antibody diluent (Dako, Carpinteria, CA, USA) and incubated overnight at 4°C. Signal was visualized with DyLight 488- or 594-conjugated secondary antibody (Thermo Fisher Scientific, Grand Island, NY, USA). Slides were coverslipped with vectashield with diamidino-2-phenylindole (DAPI; Vector Laboratories, Burlingame, CA, USA), and images were obtained and analyzed using Zeiss Imager M2 and AxioVision software (Carl Zeiss Microscopy).

### Statistical Analysis

Growth performance, histology, gene expression, and protein expression data from the *in vivo* study were analyzed using a two-way ANOVA considering chicken population (JF, ACRB, 95RAN, MRB) and environmental conditions (TN and HS) as the main effects. In case ANOVA showed significant effects, the means were compared by Tukey's multiple range test. Data from the *in vitro* study were analyzed by the Student's *t*-test. Graph Pad Prism software (version 6.00 for Windows, Graph Pad Software, La Jolla, CA, USA) was used, and data are expressed as the mean ± SEM. Means were considered statistically significant at a *P* ≤ 0.05.

## Results

### Effects of HS on Growth Performances

**Figure 1:**
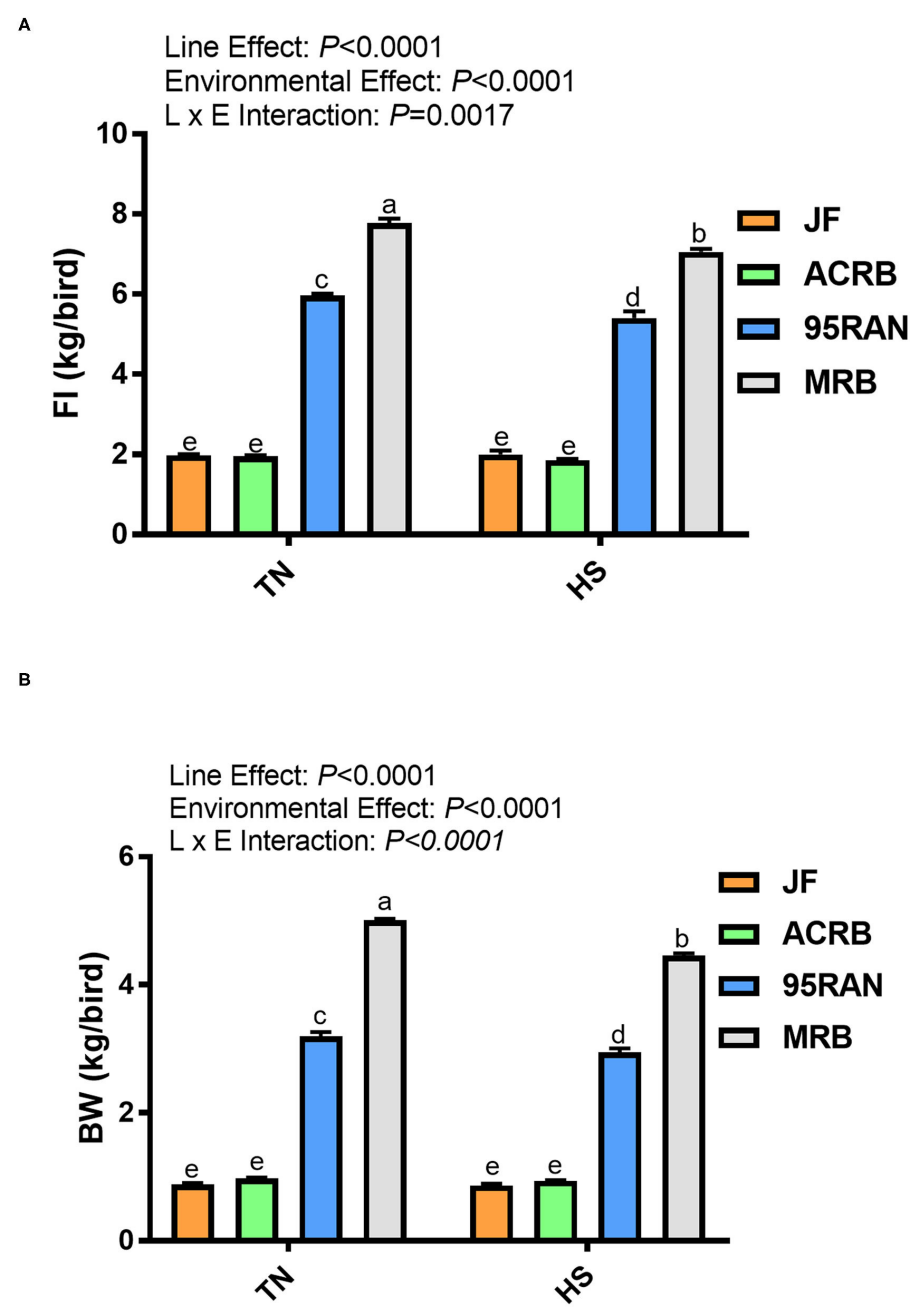
Effect of HS on feed intake **(A)** and body weight **(B)** of JF, ACRB, 95RAN, and MRB birds. Data are mean ± SEM (*n* = 3 pens/group for FI and *n* = 75 birds/group for BW). Different letters indicate a significant difference at *P* < 0.05. BW, body weight; FI, feed intake; HS, heat stress; TN, thermoneutral; JF, jungle fowl; ACRB, Athens Canadian Random Bred; 95RAN, 1995 random bred; MRB, modern random bred.

Performance of the four lines within TN and HS conditions was evaluated via comparison of BW, FI, and feed conversion ratio (FCR). Significantly higher BWs were observed for the 95RAN and MRB broilers compared to JF and ACRB in both TN and HS conditions, with the MRB having the highest BW overall (Figure 1B). However, BW for the 95RAN and MRB also decreased significantly under HS conditions compared to TN of the same population (Figure 1B). FI followed the same pattern of significance with the MRB random bred broiler having the highest FI under both TN and HS conditions (Figure 1A). The 95RAN broilers had significantly higher FI compared to JF and ACRB, but the 95RAN had lower FI compared to the MRB, and there was no difference between the JF and ACRB in either TN of HS (Figure 1A). Again, under HS conditions, FI significantly decreased in both the 95RAN and the MRB (Figure 1A). For FCR, only the main effect of the line was significant (*P* < 0.0001) while environmental treatment and interaction were not significant (*P* > 0.05). Indeed, MRB averaged 33, 52, and 78 points fewer in FCR compared to 95RAN, ACRB, and JF, respectively, confirming the tremendous effect of genetic selection in improving feed efficiency (Table 2).

**Table 2:** Effect of heat stress on the global feed conversion ratio (FCR through day 54).

	**TN**	**HS**
JF	2.349 ± 0.038	2.401 ± 0.099
ACRB	2.080 ± 0.008	2.059 ± 0.033
1995	1.890 ± 0.032	1.855 ± 0.039
2015	1.565 ± 0.022	1.598 ± 005
**Main effects**		
Environmental treatment	0.8185	
Line	<0.0001	
Interaction	0.7188	

*FCR, feed conversion ratio*.

### Effects of HS on Jejunum Morphometry

**Figure 2:**
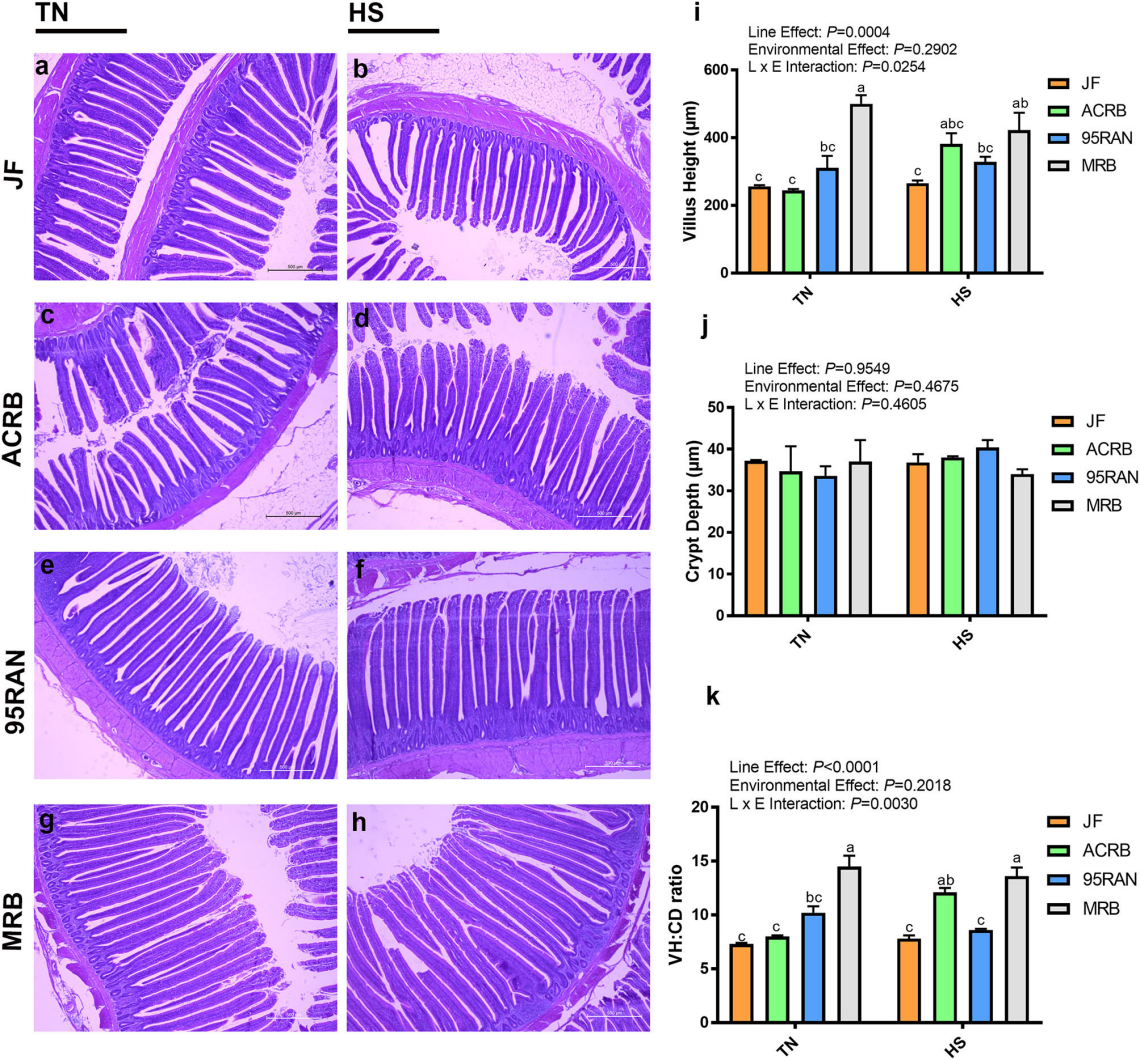
Effect of HS on intestinal morphology of JF **(a,b)**, ACRB **(c,d)**, 95RAN **(e,f)**, and MRB **(g,h)** birds. Tissue from the jejunum of TN **(a,c,e,g)** and HS **(b,d,f,h)** birds were processed and stained for histology. Data are mean ± SEM (*n* = 2/group). VH **(i)**, CD **(j)**, and VH:CD ratio **(k)** were measured and analyzed. Different letters indicate the significant difference at *P* < 0.05. VH, villus height; CD, crypt depth; HS, heat stress; TN, thermoneutral; JF, jungle fowl; ACRB, Athens Canadian Random Bred; 95RAN, 1995 random bred; MRB, modern random bred.

Histological analysis showed that jejunal VH was affected by line (*P* = 0.0004) and by line × environmental interaction (*P* = 0.0254; Figures 2a–j). Significant increased VH in MRB was seen compared to all other populations under TN conditions (*P* = 0.0030, Figures 2a–j). Under HS conditions, MRB had significantly greater VH compared to JF (Figures 2a–j). There were no significant differences in CD between the lines or when comparing environmental conditions. However, the ratio of VH:CD was affected by both line (*P* < 0.0001) and line × environmental effect (*P* = 0.0030), but not temperature (*P* = 0.2018; Figures 2a–j). Under TN conditions, the ratio of VH to CD was significantly higher in MRB compared to all other lines (Figure 2k). Under HS conditions, MRB still retained a higher ratio of VH:CD when compared to JF and 95RAN (Figure 2k). The ratio of VH to CD significantly increased from TN to HS in ACRB (Figure 2k).

### Effects of HS on the Expression of Jejunal Carbohydrate Transporters

**Figure 3:**
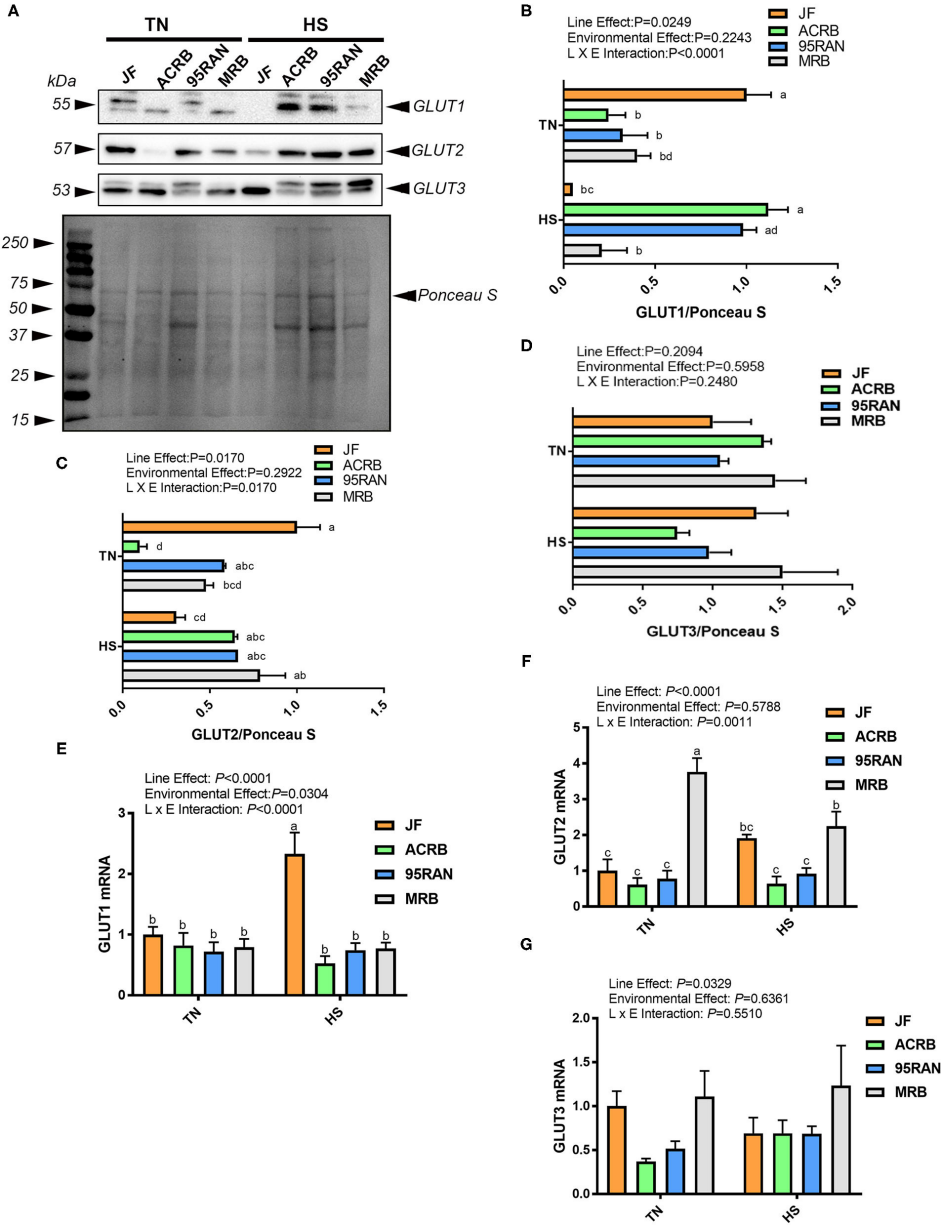
Effect of HS on jejunal expression of GLUT1, GLUT2, and GLUT3 in JF, ACRB, 95RAN, and MRB birds. Protein and RNA were extracted from tissue and analyzed by western blot **(A–D)** and qPCR **(E–G)**, respectively. Gene expression data are mean ± SEM (*n* = 6/group). Protein expression was normalized to loading via Ponceau stain (PS) **(A)**. Protein was analyzed via AlphaView software and is expressed as mean ± SEM (*n* = 4/group) with one representative blot shown **(A)**. Different letters indicate the significant difference at *P* < 0.05. GLUT, glucose transporter; HS, heat stress; TN, thermoneutral; JF, jungle fowl; ACRB, Athens Canadian Random Bred; 95RAN, 1995 random bred; MRB, modern random bred.

**Figure 4:**
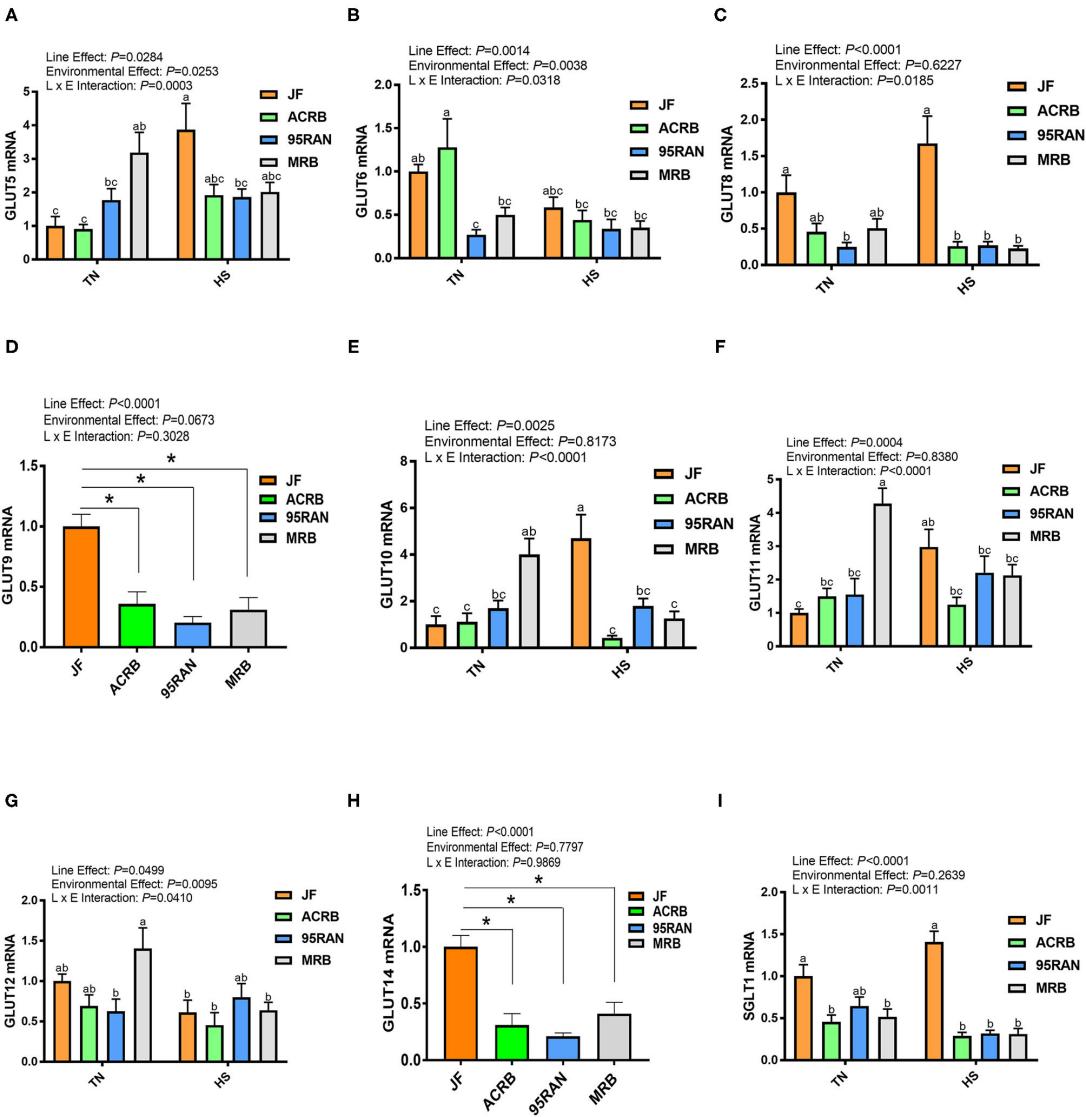
Effect of HS on the carbohydrate transporter gene expression in the jejunum of JF, ACRB, 95RAN, and MRB birds. **(A–I)** RNA was extracted from tissue and analyzed by qPCR. Gene expression data are mean ± SEM (*n* = 6/group). Different letters indicate the significant difference at *P* < 0.05 when the interaction is significant. *indicates a significance compared to JF at *P* < 0.05. GLUT, glucose transporter; HS, heat stress; SGLT1: Sodium/glucose cotransporter 1; TN, thermoneutral; JF, jungle fowl; ACRB, Athens Canadian Random Bred; 95RAN, 1995 random bred; MRB, modern random bred.

Heat stress significantly upregulated the jejunal expression of *GLUT1, GLUT5, GLUT10*, and *GLUT11* mRNA in JF, but it downregulated that of *GLUT2, GLUT10, GLUT11*, and *GLUT12* in MRB and *GLUT6* in ACRB compared to their TN counterparts (Figures 3E,F, 4A,B,E–G). The immunoblot analysis showed that jejunal GLUT1 protein levels were significantly increased in heat-stressed ACRB and 95RAN and decreased in heat-stressed JF compared to their TN counterparts (Figures 3A,B). GLUT2 protein levels were significantly increased in the jejunum of heat-stressed ACRB and decreased in heat-stressed JF compared to their TN counterparts (Figures 3A,C). Jejunal *GLUT3* mRNA abundances and protein levels remained unchanged between all chicken populations under both environmental conditions (Figures 3A,D).

Under TN conditions, the highest mRNA abundances of *GLUT2, GLUT5, GLUT10, GLUT11*, and *GLUT12* were found in the jejunum of the MRB population (Figures 3A,C, 4A,E–G). 95RAN broilers exhibited the lowest mRNA levels of *GLUT6, GLUT8*, and *GLUT9* (Figures 4B–D). Under HS conditions, the greatest expression of *GLUT1, GLUT5, GLUT8, GLUT9, GLUT10, GLUT14*, and *SGLT1* was found in the JF jejunum (Figures 3E, 4A,C–E,H,I).

**Figure 5:**
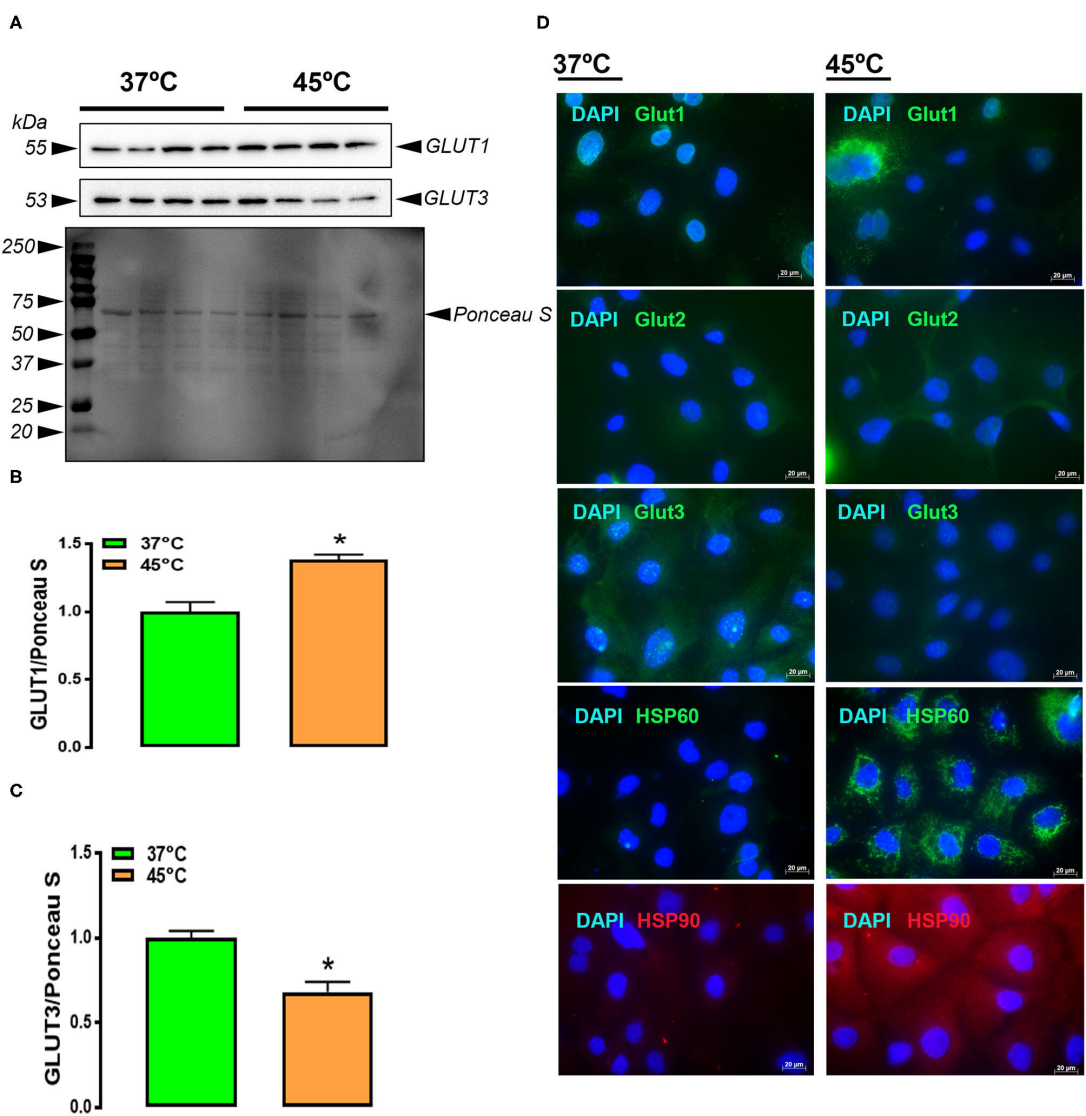
Effect of HS on glucose transporter protein expression in IPEC-J2 cell line. Protein was extracted from cells exposed to HS (45°C) or 37°C (control) and analyzed by western blot **(A–C)** and immunofluorescence **(D)**. Protein expression was normalized to loading via Ponceau stain (PS) **(A)**. Protein was analyzed via AlphaView software and is expressed as mean ± SEM (*n* = 4/group) with one blot shown **(A)**. *P < 0.05. GLUT, glucose transporter; HS, heat stress; TN, thermoneutral; DAPI: diamidino-2-phenylindole.

*in vitro* study using IPEC-J2 cell lines showed that HS exposure induced the expression of HSP60 and HSP90 as demonstrated by immunofluorescence staining (Figure 5D), confirming that the cells are stressed. Both immunoblot analysis and immunofluorescence staining showed that HS increased GLUT1 and decreased GLUT3 protein levels in the IPEC-J2 cells (Figures 5A–D).

### Effects of HS on the Expression of Jejunal Amino Acid Transporters

**Figure 6:**
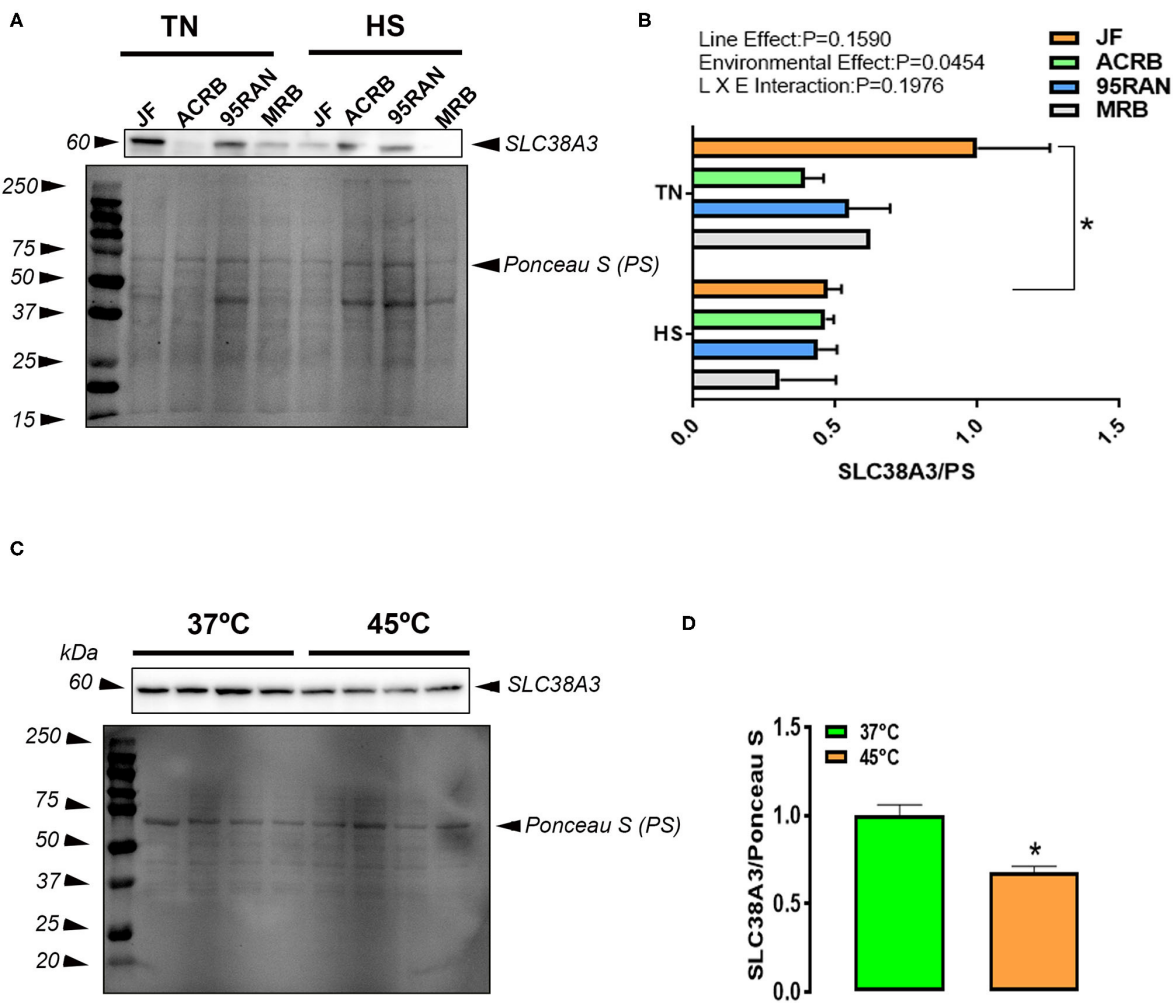
Amino acid transporter protein expression in the jejunum of JF, ACRB, 95RAN, and MRB birds under TN and HS and in IPEC-J2 cell line. Protein was extracted from tissue and cells exposed to HS (45°C) or to 37°C (control) and analyzed by western blot **(A–D)**. Protein expression was normalized to loading via Ponceau stain (PS) **(A,C)**. Protein was analyzed via AlphaView software and is expressed as mean ± SEM (*n* = 4/group) for IPEC-J2 cells and (*n* = 4/group) for tissue. *indicate a significant difference compared to TN conditions for bird populations or 37°C for IPEC-J2 cell line at *P* < 0.05. HS, heat stress; TN, thermoneutral.

Western Blot analysis of amino acid transporter showed that HS significantly decreased SLC38A3 protein levels in the jejunum of JF but not in the other bird populations (Figures 6A,B). Under TN conditions, jejunal expression of SLC38A3 protein is higher in JF compared to the other populations (Figures 6A,B). Under HS conditions, however, the levels of SLC38A3 protein remained unchanged between all tested birds (Figures 6A,B). In IPEC-J2 cells, SLC38A3 protein expression was significantly downregulated by HS exposure (Figures 6C,D).

**Figure 7:**
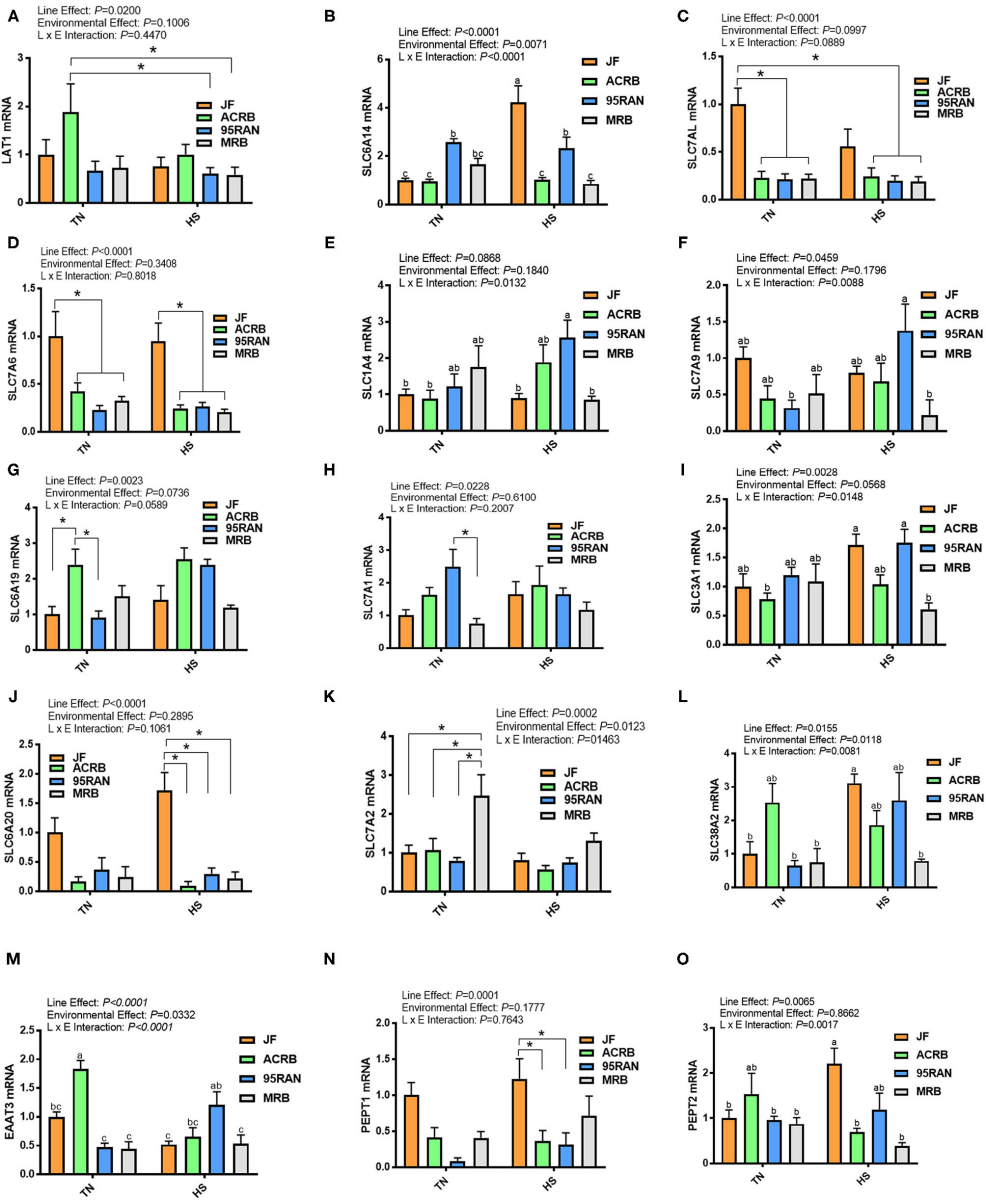
Amino acid transporter gene expression in the jejunum of JF, ACRB, 95RAN, and MRB birds under TN and HS. **(A–O)** RNA was extracted from tissue and analyzed by qPCR. Gene expression data are mean ± SEM (*n* = 6/group). * and different letters indicate a significant difference at *P* < 0.05. LAT1, L-type amino acid transporter 1; EEAT3, excitatory amino acid transporter 3; PEPT, peptide transporter; SLC, Solute carrier family; HS, heat stress; TN, thermoneutral; JF, jungle fowl; ACRB, Athens Canadian Random Bred; 95RAN, 1995 random bred; MRB, modern random bred.

Heat stress significantly increased the mRNA levels of *SLC6A14, SLC38A2*, and *PEPT2* gene in the jejunum of JF (Figures 7B,L,O), *SLC7A9*, and *EAAT3* genes in 95RAN (Figures 7F,M), and significantly decreased that of *EAAT3* gene in ACRB (Figure 7M). Under TN conditions, the highest mRNA abundances of *SLC7AL* and *SLC7A6* were found in the jejunum of JF (Figures 7C,D), *SLC6A19* and *EAAT3* in ACRB (Figures 7G,M), *SLC7A1* in 95RAN (Figure 7H) and *SLC1A4* and *SLC7A2* in MRB (Figures 7E,K). Under HS conditions, however, JF jejunum exhibited greater expression of *SLC6A14*, SLC7A6, *SLC6A20, PEPT1*, and *PEPT2* (Figures 7B,D,J,N,O). 95RAN birds on the other hand contained a higher expression of jejunal *SLC1A4, SLC7A9*, and *EAAT3* genes (Figures 7E,F,M). HS significantly down regulated SLC3A1 expression in MRB compared to JF and 95RAN (Figure 7I).

### Effects of HS on the Expression of Jejunal FATs

**Figure 8:**
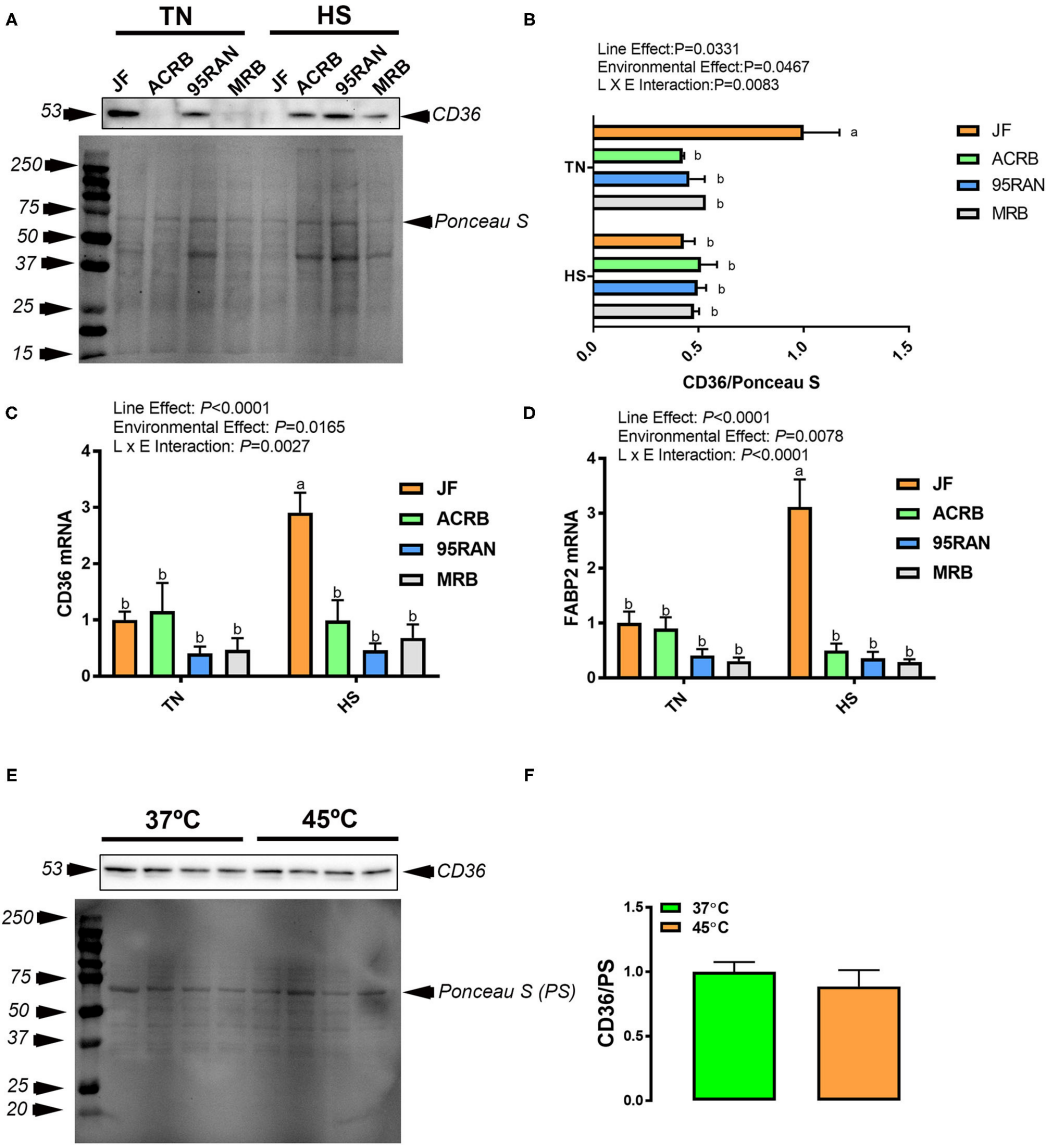
Effect of HS on the expression of fatty acid transporters in the jejunum of JF, ACRB, 95RAN, and MRB birds and in IPEC-J2 cell line. Protein and RNA were extracted from jejunal tissue and analyzed by western blot **(A,B)** and qPCR **(C,D)**, respectively. Protein from cells was also analyzed by immunoblot **(E,F)**. Gene and protein expression data are mean ± SEM (*n* = 4–6/group). Protein expression was normalized to loading via Ponceau stain (PS). Protein was analyzed via AlphaView software and one representative blot is shown. Different letters indicate a significant difference at *P* < 0.05 when the interaction effect is significant. CD36, the cluster of differentiation 36; FABP, fatty acid-binding protein; HS, heat stress; TN, thermoneutral; JF, jungle fowl; ACRB, Athens Canadian Random Bred; 95RAN, 1995 random bred; MRB, modern random bred.

Immunoblot analyses showed that HS significantly reduced CD36 protein levels in the jejunum of JF, but not in the other bird populations (Figures 8A,B). However, mRNA abundances of both *CD36* and *FABP2* were significantly increased in heat-stressed JF compared to their TN counterparts (Figures 8C,D). HS exposure did not elicit any changes to CD36 protein expression in the IPEC-J2 cell line (Figures 8E,F).

Under TN conditions, jejunal *CD36* and *FABP2* mRNA abundances remained unchanged between all chicken populations; however, CD36 protein levels were significantly higher in JF (Figures 8A–D). Under HS exposure, jejunal CD36 protein expression did not differ between all tested bird populations, however, *CD36* and *FABP2* gene expressions were higher in JF (Figures 8A–D).

## Discussion

With the aim to fulfill the increasing demand for poultry meat around the world, genetic selection has led to spectacular achievements in terms of growth rate, feed efficiency, breast yield, and reduction of market age (Thiruvenkadan and Prabakaran, 2017). In the current study, the performance of 54 d-old broilers represented genetics of the 1990s (95RAN) and 2015 (MRB) exhibited a much higher growth rate, and lower FCR compared to the broilers from the 1950s (ACRB) and the ancestral wild-type JF, confirming the aforesaid advancements. However, these signs of progress have been associated with unintended, undesirable consequences, including high sensitivity to HS, whose adverse effects on poultry production and welfare are well-documented in the literature. These effects are expected to only get worse in the coming years due to the steady increase in environmental temperature (Wasti et al., 2020; Perini et al., 2021). Indeed, HS has been reported to compromise gut health leading to decreased nutrient absorption in broiler chickens (Goel et al., 2021). In this context, the current study aimed to evaluate the differences in response to HS of four chicken populations representative of eras in the history of a selection of the modern broilers, the JF, the ACRB, the 95RAN, and the MRB. Response to HS was evaluated via analysis of nutrient transport machinery in the jejunum of broiler chickens. To the best of our knowledge, the current study provides the first comparison of the mRNA gene expression and some of the encoded proteins involved in the transport of nutrients (carbohydrates, amino acids, and fatty acids) along with histological evaluation of the jejunum, between the four genetic lines.

The uptake of carbohydrates from the intestinal lumen is crucial to sustaining a constant energy supply and is influenced by both luminal and apical membrane digestion (Sklan et al., 2003). Results from this study suggest that differences in glucose transporter expression in the jejunum depend on line, or the representative genetics over time, and therefore, these differences have the potential to be due to, or the result of, genetic differences. The results also suggest that the expression and presence of glucose transporters in the gut may play a role in HS intolerance or resistance among these different populations. This is evident by the increased gene expression in MRB of *GLUT2*, involved in the transport of carbohydrates, such as glucose, galactose, and fructose across the basolateral membrane, *GLUT5*, a fructose transporter across the brush border membrane (Uldry and Thorens, 2004), *GLUT10*, and *GLUT11* as compared to JF and ACRB under TN conditions. In line with these, a study conducted by Miska and Fetterer (2019) showed *GLUT2* and *GLUT5* mRNA levels to be higher in modern fast-growing Ross as compared to the slow-growing ACRB chickens. The increased gene expression of these transporters in modern birds may emphasize the importance of carbohydrate transporters in the fast-growing chickens providing them with greater capacity to absorb sugars. Conversely, GLUT2 protein expression was shown to be higher in JF compared to ACRB and MRB under TN conditions. GLUT2 significantly decreased in JF under HS conditions while significantly increased in ACRB. The significant increase in protein levels of GLUT2 seen in ACRB and the lack of effect on more modern populations compared to the heat-tolerant JF may be a product of their propensity for better growth performance and also contribute to their intolerance of HS conditions. The heat-stressed MRB chickens also showed a decrease in gene expression of some GLUTs, such as *GLUT10* and *GLUT12* as compared to their TN counterparts, being in line with previous studies reporting a decrease of the jejunum *GLUT2* (Al-zghoul et al., 2019) and ileum *GLUT1* (Habashy et al., 2017b) by applying a chronic HS of 35°C for 7 and 12 days, respectively. These results suggest that the uptake of glucose and galactose was compromised in heat-stressed chickens which may explain the reduction of BW at day 54 in heat-stressed MRB chickens as compared to their TN counterparts. However, the reduction of BW at day 54 was not associated with a decreased VH:CD ratio previously observed in heat-stressed chickens (Song et al., 2017) and considered as a measure of the absorptive capacity of the gut. The lack of effect on intestinal histology may be a sign of recovery or adaptation after 26 days of cyclic HS. On the other hand, heat-stressed JF showed an increase of *GLUT1, GLUT5, GLUT10*, and *GLUT11* gene expression as compared to their TN counterparts, and greater mRNA levels of *GLUT1, GLUT8, GLUT9, GLUT10*, and *SGLT1* compared to the rest of the lines. It is difficult to make an across study comparative conclusion of how differences in growth capacity among chicken populations affect their response to HS due to the lack of research comparing the ancestor JF with other lines characterized by higher growth rate. However, the results obtained in the current study suggest either JF to be more resistant to HS, or it is nutrient transport machinery to present a more robust response to HS challenging conditions. Granted, this conclusion only bears true if the nutrient transporter machinery responses seen in JF in this study correspond to increased absorption, which was not measured, and if that corresponding absorption in the gut aids in JF resistance to HS.

In 2013, Pearce et al. showed an increased incidence of hyperglycemia and an increase in the expression of ileal GLUTs in heat-stressed pigs. Interestingly, the IPEC-J2 expression of GLUT1 and GLUT3 protein followed the same trends as in chickens (at least the ACRB and 95RAN for GLUT1 and ACRB for GLUT2), indicating that HS had a direct effect on jejunal carbohydrate transporters. Given that the functionality of these transporters is often dependent on their cellular location, further investigation is needed to determine the cellular location and provide a potential explanation for possible trends in expression.

Several amino acid transport systems are expressed on both the apical brush border membrane and basolateral side of the small intestine epithelium (Dave et al., 2004) to bring the amino acids from the gut lumen into the enterocytes, and from inside of the enterocyte to the vascular supply or vice versa, respectively. Results of the current study suggest amino acid transporters (AATs) to be less responsive to HS when compared to carbohydrate transporters. When comparing significant differences seen among amino acid transporters only, the effect on gene expression seems to be more dependent on line than environment for populations other than the MRB. Indeed, as compared to other populations under TN conditions, ACRB birds exhibited greater gene expression of *LAT1* and *SLC38A2*, which are present on the basolateral membrane, and *SLC6A19, EAAT3*, and *PEPT2* which encode brush border AATs. However, JF expressed greater *SLC7A6*, present on the basolateral membrane, and *PEPT1*, a brush border AAT, as compared to the rest of TN populations. *PEPT1* and *EAAT3* have been previously reported to be higher in the small intestine of birds selected for low compared to high juvenile BW (Mott et al., 2008). Similarly, *SLC7A1, SLC7A4, SLC3A1, SLC7A6*, and *SLC7A7* gene expression was greater in the intestine of a Chinese slow-growing bird (Wenshi Yellow Feathered Chick; WYFC) as compared to a commercial fast-growing broiler (White Recessive Rock Chick; WRRC) (Zeng et al., 2011). Moreover, a recent study conducted by Miska and Fetterer (2019) showed *SLC1A4, SLC6A14*, and *SLC6A19* to be greater in the intestine of ACRB as compared to ROSS broilers. The reason behind this increase is not fully elucidated, but it is possible that AATs of slow-growing lines are less efficient than those of fast-growing lines, such that a higher number of AATs is required to transport a smaller amount of nutrients. As for HS, although its negative effects on nutrient digestibility and absorption are well-described, only a small number of studies have reported HS effects on AATs. In a recent study, Al-zghoul et al. (2019) reported *SLC7A7* to be upregulated and *SLC7A1* to be downregulated by HS; however, this is in contrast with other studies showing no effect on these AATs (Sun et al., 2015; Yi et al., 2016; Song et al., 2017). This discrepancy may be attributed to the heterogeneity of experimental conditions, such as lines, feed, age, duration, and severity of HS. Results of the current study showed differing responses to HS among lines. Although HS decreased *SLC6A14* in MRB and *EAAT3* in ACRB, it increased *SLC6A14* and *SLC38A2* in JF and *SLC7A9, SLC6A19*, and *EAAT3* in 95RAN. The jejunum expression of the *EAAT3* gene was also enhanced in ROSS broilers exposed to 35–39°C for 1–5 days (Santos et al., 2019). This increase of *EAAT3* may indicate a greater need for aspartate and glutamate uptake acting as the primary fuel for enterocytes, to maintain intestinal permeability and enterocyte number negatively affected by heat-induced tissue damage (Goel et al., 2021). Another interesting finding in this study was the lack of HS effect on the expression of AAT in MRB. Indeed, a decrease was seen only for *SLC6A14* for MRB while all others remained unchanged. This may suggest that MRB may retain their amino acid absorption regardless of environmental stressors, such as HS, due to the effects of high-performance selection the MRB has gone through over time. However, more research is needed to confirm any direct effects of selection on these AAT and the role HS plays in the dynamics of genotype and environment interactions.

Protein expression of AATs showed a main effect of temperature on decreasing SLC38A3, which seems to be driven by the effects in JF. Similarly, IPEC-J2 cells subjected to HS conditions also showed significantly decreased SLC38A3 protein. It is possible that an IPEC-J2 model for HS effects on AAT in the gut better represents ancestral JF than more modern populations. However, further analysis is needed to fully characterize IPEC-J2 as a model for JF under HS. These results also point to the potential need for specific *in vitro* cell models per population.

Heat stress did not affect protein expression of fatty acid translocator CD36 in ACRB, 95RAN to MRB; however, JF showed a decrease under HS compared to TN conditions. This lack of response from more modern populations coupled with the JF decreased expression of CD36 under HS may indicate a possible role for decreased fatty acid absorption in resistance to HS and that a lack of response in this translocation may be a result of selection for higher growth performance.

The levels of mRNA for both *FABP2* and *CD36* showed a similar pattern of expression whereby there were no differences among the four populations under TN conditions. HS increased the expression of both FATs in JF compared to the rest of the populations. However, previous studies reported that *FABP*, which mediates the uptake of long-chain fatty acids into enterocytes, was downregulated irrespective of exposure time and intensity of stress. Indeed, *FABP* gene expression was reduced in male Arbor Acres broilers exposed to 32°C from 35 to 42 days of age (Sun et al., 2015), and male Cobb500 subjected to 35°C from 14 to 26 days of age (Habashy et al., 2017b). Similar results were reported with *CD36*, where jejunal expression was shown to be reduced in heat-stressed fast-growing broilers (Sun et al., 2015). The inconsistency between the gene expression results for FATs obtained in the current study and previous ones may be attributed to differences in the temperature, duration of HS, and specific strains of birds used. Moreover, *CD36* gene expression did not show the same pattern as protein expression in the current study. A similar disparity was seen in several other transporters measured and is not surprising, as the correlation between protein concentrations and the corresponding mRNA levels has been previously investigated and established to be 20–40% (Pascal et al., 2008; Zapalska-Sozoniuk et al., 2019) due to several mechanisms such as the rate of mRNA degradation/turnover and efficiency of translation.

## Conclusion

This study is the first to describe differences in the expression of genes, and some of the encoded proteins, that play a role in jejunal nutrient transport between three broiler populations characterized by different growth (slow, moderate, and rapid) rates and in their ancestor wild JF birds. The obtained results seemingly show that chronic HS alters the jejunal carbohydrate, rather than fatty acid and amino acid, transporters and that differences in these transporters between the populations may be a result of, or mechanism behind, the phenotypic differences that exist. Understanding the molecular mechanisms behind these differences and their potential relationship to selection is of great interest; however, further in-depth investigation is needed and warranted based on these findings.

## Data Availability Statement

The original contributions presented in the study are included in the article/supplementary material, further inquiries can be directed to the corresponding author/s.

## Ethics Statement

The animal study was reviewed and approved by University of Arkansas.

## Author Contributions

SD conceived and designed the study. TT, EG, SO, NBA, and SD conducted the *in vivo* experiments. NA and AR performed the molecular analyses and analyzed the data. LB performed the IF images. NA and AR wrote the first draft. SD edited and corrected the paper with a critical review by all authors.

## Funding

This study was supported by a grant from the Arkansas Division of Agriculture, Animal Health Awards (to SD and SO) and from the USDA-AFRI Sustainable Agriculture Systems (2019-69012-29905) to SD. The Arkansas Division of Agriculture and USDA-AFRI had no role in conducting the research, generating the data, interpreting the results, or writing the manuscript.

## Conflict of Interest

The authors declare that the research was conducted in the absence of any commercial or financial relationships that could be construed as a potential conflict of interest.

## Publisher's Note

All claims expressed in this article are solely those of the authors and do not necessarily represent those of their affiliated organizations, or those of the publisher, the editors and the reviewers. Any product that may be evaluated in this article, or claim that may be made by its manufacturer, is not guaranteed or endorsed by the publisher.
